# AS-TBR: An Intrusion Detection Model for Smart Grid Advanced Metering Infrastructure

**DOI:** 10.3390/s25103155

**Published:** 2025-05-16

**Authors:** Hao Ma, Yifan Fan, Yiying Zhang

**Affiliations:** College of Artificial Intelligence, Tianjin University of Science and Technology, Tianjin 300222, China; mahao164ok@163.com (H.M.); fanyifan@mail.tust.edu.cn (Y.F.)

**Keywords:** advanced metering infrastructure, intrusion detection, transformer, BiGRU, ResNet, imbalanced data

## Abstract

Advanced Metering Infrastructure (AMI), as a critical data collection and communication hub within the smart grid architecture, is highly vulnerable to network intrusions due to its open bidirectional communication network. A significant challenge in AMI traffic data is the severe class imbalance, where existing methods tend to favor majority class samples while neglecting the detection of minority class attacks, thereby undermining the overall reliability of the detection system. Additionally, current approaches exhibit limitations in spatiotemporal feature extraction, failing to effectively capture the complex dependencies within network traffic data. In terms of global dependency modeling, existing models struggle to dynamically adjust key features, impacting the efficiency and accuracy of intrusion detection and response. To address these issues, this paper proposes an innovative hybrid deep learning model, AS-TBR, for AMI intrusion detection in smart grids. The proposed model incorporates the Adaptive Synthetic Sampling (ADASYN) technique to mitigate data imbalance, thereby enhancing the detection accuracy of minority class samples. Simultaneously, Transformer is leveraged to capture global temporal dependencies, BiGRU is employed to model bidirectional temporal relationships, and ResNet is utilized for deep spatial feature extraction. Experimental results demonstrate that the AS-TBR model achieves an accuracy of 93% on the UNSW-NB15 dataset and 80% on the NSL-KDD dataset. Furthermore, it outperforms baseline models in terms of precision, recall, and other key evaluation metrics, validating its effectiveness and robustness in AMI intrusion detection.

## 1. Introduction

Advanced Metering Infrastructure (AMI), as a core component of the smart grid, enables the precise collection and analysis of electrical data, supporting key functions such as time-of-use pricing and fault detection [1]. The introduction of AMI not only improves the operational efficiency of power grids but also provides technical support to address complex electricity demands.

However, as AMI relies on bidirectional communication to enable interactions between users and grid systems, it introduces potential risks such as denial-of-service attacks and false data injection, despite enhancing the efficiency of grid regulation [2]. Such intrusions may lead to power outages or system failures, threatening the stability of smart grids and compromising public safety. Therefore, ensuring the security of AMI has become a critical task in safeguarding the reliability of power grids and societal safety.

Intrusion Detection Systems (IDS), as a critical line of defense in smart grids, monitor abnormal activities within networks or systems to detect potential intrusion attempts and take countermeasures. By addressing threats at an early stage, IDS can curb the spread of attacks and enhance the security of AMI systems [3]. However, intrusion detection systems applied to Advanced Metering Infrastructure (AMI) still face several challenges.

First, the class imbalance in network traffic data causes existing models to prioritize the detection of majority class samples while neglecting the recognition of minority class samples. This increases the risk of missed detections and undermines the overall reliability and security of the detection system. Second, power grid data typically contain complex spatiotemporal dependencies, which traditional feature extraction techniques fail to fully exploit. In this study, the spatial features in the network traffic data refer to attributes such as the source IP address, destination IP address, port numbers, and packet sizes, which reflect the structural characteristics of network communications. The temporal features include the timestamps of packet transmissions and the dynamic changes in traffic volume over time, capturing the time-varying patterns of network behavior. By jointly considering these spatial and temporal dimensions, the model can better identify complex intrusion patterns that are difficult to detect when examining spatial or temporal features alone. This limits the system’s ability to identify complex attack patterns. Furthermore, existing models exhibit deficiencies in capturing global dependencies, particularly in extracting temporal sequence features, where they struggle to dynamically adjust critical features. These limitations affect the efficiency and accuracy of detection and response.

To address the aforementioned challenges, this paper proposes a hybrid deep learning model tailored for AMI, named TBR-IDS, aiming to enhance the reliability and accuracy of intrusion detection. To mitigate the issue of minority class misdetection caused by class imbalance in network traffic data, the Adaptive Synthetic Sampling (ADASYN) technique is introduced. By dynamically balancing the data distribution, ADASYN improves the model’s ability to detect minority class attacks, thereby enhancing the overall robustness of the detection system.

To effectively extract complex spatiotemporal features from AMI traffic data and achieve precise global dependency modeling, the proposed model integrates multiple feature extraction modules. Specifically, the Transformer encoder is employed to capture global temporal dependencies, BiGRU is used to model bidirectional temporal features, and ResNet is utilized to extract deep spatial features. This multi-level feature extraction architecture enables the model to more effectively capture global dependencies and dynamically adjust the weights of critical features during feature extraction, significantly improving the efficiency and accuracy of detection and response.

Based on the integration of the above methods and architecture, the model is named AS-TBR. The main contributions of this paper are as follows:

- To address the issue of class imbalance in network traffic data, the Adaptive Synthetic Sampling (ADASYN) technique is introduced. By dynamically sampling minority class instances, the model’s performance in detecting minority class samples is improved, enhancing the robustness of intrusion detection.
- The Transformer encoder is employed to capture global temporal dependencies, leveraging the self-attention mechanism to dynamically adjust the weights of critical features, thereby further improving the detection accuracy and response speed of the model.
- A spatiotemporal feature extraction module combining BiGRU and ResNet is designed. BiGRU is used to model bidirectional temporal features, while ResNet is utilized to extract deep spatial features, enhancing the model’s ability to recognize complex attack patterns.

## 2. Related Work

Existing methods for intrusion detection in Advanced Metering Infrastructure (AMI) can be broadly divided into two categories: machine learning-based and deep learning-based approaches. Machine learning methods offer a certain degree of interpretability but are significantly limited in recognizing complex patterns. In contrast, deep learning methods excel in handling high-dimensional data but still face challenges in computational efficiency and detecting minority class samples [4].

In AMI intrusion detection, traditional machine learning methods often employ algorithms such as Random Forest (RF), Support Vector Machine (SVM), and Decision Tree (DT). For instance, ref. [5] proposed an SVM model combined with Naive Bayes feature transformation and embedding functions. While it performed well on the UNSW-NB15 dataset, it was limited to binary classification tasks and struggled with multi-class attacks. In [6], a multi-class SVM combined with mutual information-based feature selection achieved improved detection rates, but the multi-classifier structure resulted in extended training times and lower computational efficiency. Addressing class imbalance, ref. [7] proposed an RF-GBDT model combining Random Forest and Gradient Boosted Decision Trees, which enhanced minority class detection. However, the high computational complexity of the ensemble model and lengthy training times hinder practical applications.

In contrast, deep learning methods are more flexible for AMI intrusion detection. For example, ref. [8] employed a CNN-based intrusion detection model that improved detection efficiency through weight sharing, but the unoptimized structure limited model performance. To extract temporal features, ref. [9] introduced an RNN model to capture communication dependencies, but the vanishing gradient problem during training reduced its efficiency. Ref. [10] proposed an LSTM-based improvement of the RNN model, achieving 84.83% accuracy on the NSL-KDD dataset, yet it still struggled with long-sequence data. A BiLSTM model introduced in [11] improved detection and recall rates but suffered from slow training speeds. Meanwhile, ref. [12] optimized temporal modeling using GRU, improving computational efficiency, but GRU’s bidirectional modeling might miss key information related to the current timestep. To address the issue of class imbalance, Giuseppina [13] proposed a GAN-based intrusion detection model, combining GAN and CNN to generate attack events and mitigate class imbalance. This model improved detection accuracy, achieving 93.29% accuracy on the KDD99 dataset. Similarly, ref. [14] applied an AE-LightGBM model in SDN networks, addressing data redundancy and imbalance through SMOTE oversampling and AE-based feature extraction, achieving 99.90% and 99.70% accuracy on the KDD CUP99 and NSL-KDD datasets, respectively. However, this approach faced challenges with long training times due to the use of gradient descent algorithms.

While deep learning methods demonstrate advantages in feature extraction and adaptability, current models still face challenges in computational efficiency, long-sequence dependency handling, and minority class detection.

## 3. Proposed Method

### 3.1. ADASYN

ADASYN (Adaptive Synthetic Sampling) is an adaptive oversampling method designed for imbalanced datasets. The core idea is to generate more minority class samples in complex decision regions, dynamically adjusting the sample distribution, which enhances the classifier’s ability to recognize minority class instances. Compared to traditional oversampling methods, ADASYN focuses on generating samples in the complex boundary regions, helping the model better learn the decision boundaries between minority and majority classes, thereby improving classification performance. The steps of ADASYN are as follows:

(1) K-Nearest Neighbor Calculation: For each minority class sample, compute its K-nearest neighbors and count the number of majority class samples among these neighbors.

(2) Determine the Number of Samples to Generate: Based on the difficulty of the minority class sample, determine the number of synthetic samples to generate. Regions with higher difficulty generate more new samples, while those with lower difficulty generate fewer. The difficulty $r_{i}$ is defined as

(1) $$r_{i}=\frac{M_{i}}{K}$$

where $r_{i}$ represents the proportion of majority class samples around the minority class sample $x_{i}$, $M_{i}$ is the number of majority class neighbors around $x_{i}$, and *K* is the number of nearest neighbors.

(3) Generate synthetic samples: For each minority class sample, a random sample is selected from its K-nearest neighbors, and new samples are generated using linear interpolation. The generation formula for synthetic samples is

(2) $$x_{new}=x_{i}+\lambda \cdot \left(x_{neighbor}-x_{i}\right)$$

where *λ* is a random number in the range [0, 1].

(4) Update the dataset: Add the generated synthetic samples to the original dataset, thereby increasing the final number of minority class samples.

### 3.2. Transformer Encoder

Transformer is a deep learning model based on the attention mechanism, consisting of an encoder and a decoder. The encoder is responsible for receiving the input sequence and generating representations containing contextual information, while the decoder generates the target sequence based on the output of the encoder. In the task of network intrusion detection, only the classification of input data is required, so the encoder part is used to extract features. The structure of the encoder is shown in the figure.

To enable the model to understand the positional information in the input sequence, Transformer introduces positional encoding. Positional encoding is generated using sine and cosine functions, allowing the model to capture the relative positional information within the input sequence. The formula is

(3) $$PE_{pos,2i}=sin\left(\frac{pos}{{10,000}^{\frac{2i}{d_{model}}}}\right)$$

(4) $$PE_{pos,2i+1}=cos\left(\frac{pos}{{10,000}^{\frac{2i}{d_{model}}}}\right)$$

where pos represents the position, *i* is the dimension index, and $d_{model}$ is the dimensionality of the model.

The core of the encoder is the multi-head attention mechanism. This mechanism extracts features by calculating the dependencies between different positions in the input sequence. The attention calculation formula is as follows:

(5) $$\mathrm{Attention}\left(Q,K,V\right)=\mathrm{softmax}\left(\frac{QK^{T}}{\sqrt{d_{k}}}\right)V$$

*Q*, *K*, and *V* represent the query, key, and value, respectively, and $d_{k}$ is the dimensionality of the key. The output of the multi-head attention can be expressed as

(6) $$\begin{array}{c} \mathrm{MultiHead}\left(Q,K,V\right)=\\ \mathrm{Concat}\left({head}_{1},\ldots ,{head}_{h}\right)W^{O} \end{array}$$

where each attention head is calculated as

(7) $${head}_{i}=Attention\left(QW_{i}^{Q},KW_{i}^{K},VW_{i}^{V}\right)$$

Each encoder layer is followed by a feed-forward neural network (FFN) after the multi-head attention mechanism. The formula for the FFN is as follows:

(8) $$FFN\left(x\right)=max\left(0,xW_{1}+b_{1}\right)W_{2}+b_{2}$$

*b* is the weight matrix, and *W* is the bias vector. The design of the feed-forward network further enhances the feature extraction capability.

To ensure stability during model training, the encoder introduces residual connections and layer normalization after each sublayer. The formula for the residual connection is as follows:

(9) $$Output=\mathrm{LayerNorm}\left(x+Sublayer\left(x\right)\right)$$

This helps prevent gradient vanishing, while layer normalization accelerates the convergence of the model.

**Figure 1:**
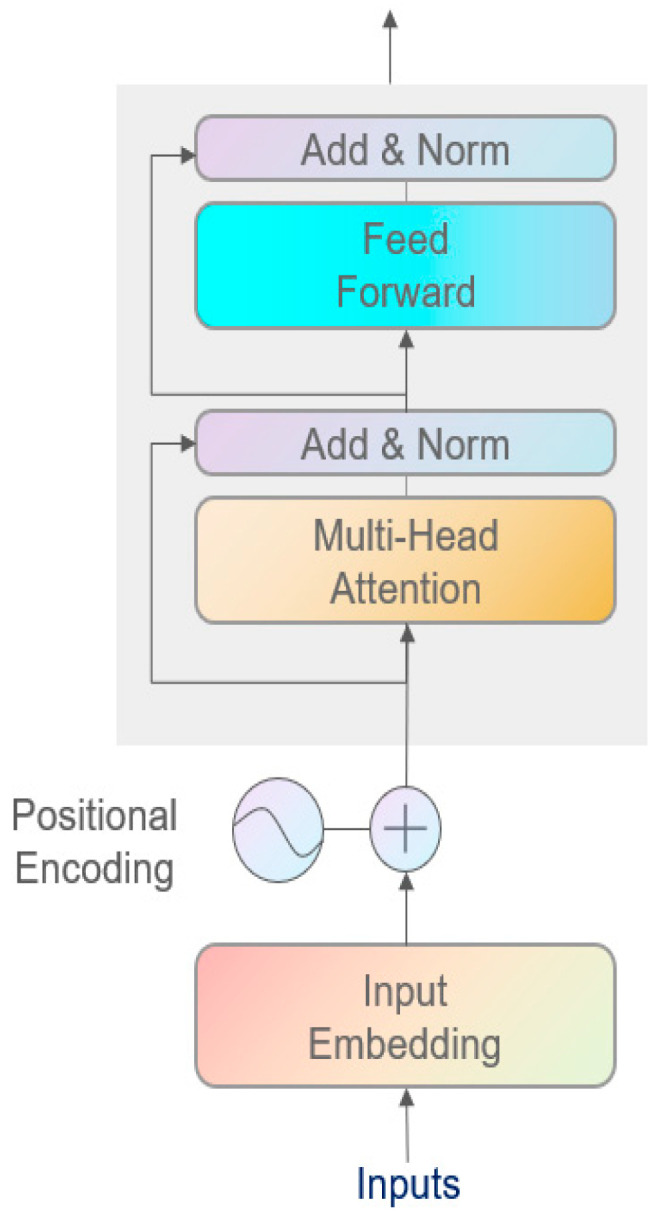
Transformer encoder structure diagram.

The structure of the Transformer encoder is shown in Figure 1. In this work, the Transformer encoder is employed to capture the long-range temporal dependencies in network traffic sequences, enabling the model to learn global timing patterns crucial for distinguishing between normal and anomalous activities.

### 3.3. BiGRU

**Figure 2:**
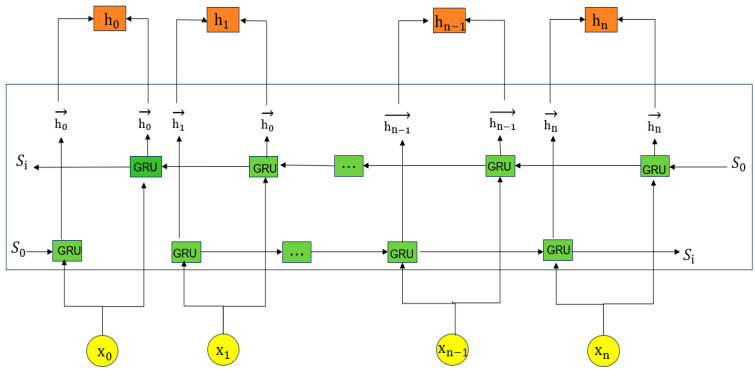
BiGRU structure.

The Bidirectional Gated Recurrent Unit (BiGRU) is an improved RNN structure that captures forward and backward sequence dependencies through its bidirectional architecture, addressing the issue of information loss in long sequences. The model structure is shown in Figure 2. BiGRU consists of two GRU units: one processes forward information, while the other handles backward information, enabling the model to obtain complete contextual information at each time step.

For each time step *ttt*, the calculation formula for the update gate $z_{t}$ is as follows:

(10) $$z_{t}=\sigma \left(W_{z}x_{t}+U_{z}h_{t-1}\right)$$

*σ* is the Sigmoid activation function, $W_{Z}$ and $U_{Z}$ are the weight matrices, $X_{t}$ is the current input, and $h_{t-1}$ is the hidden state from the previous time step. The update gate determines the extent to which information from the previous hidden state is retained.

The reset gate $r_{t}$ controls the extent to which information from the previous hidden state is discarded when calculating the candidate state. The calculation formula is as follows:

(11) $$r_{t}=\sigma \left(W_{r}x_{t}+U_{r}h_{t-1}\right)$$

The new candidate state ${\tilde{h}}_{t}$ combines the current input and the reset previous state. Its calculation formula is as follows:

(12) $${\tilde{h}}_{t}=tanh\left(W_{h}x_{t}+U_{h}\left(r_{t}⊙h_{t-1}\right)\right)$$

⊙ represents element-wise multiplication, ensuring that the reset gate can effectively control the contribution of the previous state. The final hidden state $h_{t}$ is a weighted combination of the previous hidden state and the current candidate state. The update formula is as follows:

(13) $$h_{t}=\left(1-z_{t}\right)⊙h_{t-1}+z_{t}⊙{\tilde{h}}_{t}$$

This design enables BiGRU to effectively transmit important information between time steps while avoiding information loss. The BiGRU module is utilized to model bidirectional local temporal dependencies, allowing the model to effectively capture the subtle and sequential changes in network traffic, which are essential for accurately recognizing intrusion behaviors over short time periods.

### 3.4. Residual Network

ResNet (residual network) is a type of deep convolutional neural network that alleviates the problems of gradient vanishing and information loss in deep networks through “residual connections,” thereby enhancing feature extraction capabilities. Its core use is to simplify the learning process and improve the network’s representational power and training efficiency by connecting the input and output across layers through skip connections.

In residual learning, for a vector $x_{ij}$ from the preprocessed feature matrix T, the model learns the differential features through the residual function $F\left(x_{ij}\right)=H\left(x_{ij}\right)-x_{ij}$, which can be expressed as $H\left(x_{ij}\right)=F\left(x_{ij}\right)+x_{ij}$ to represent the learning of the original features. If the residual is 0, the stacked layers become an identity mapping without degradation. In practice, however, the residual is non-zero, enabling the model to learn new feature representations based on the input features, thereby enhancing the network’s performance.

**Figure 3:**
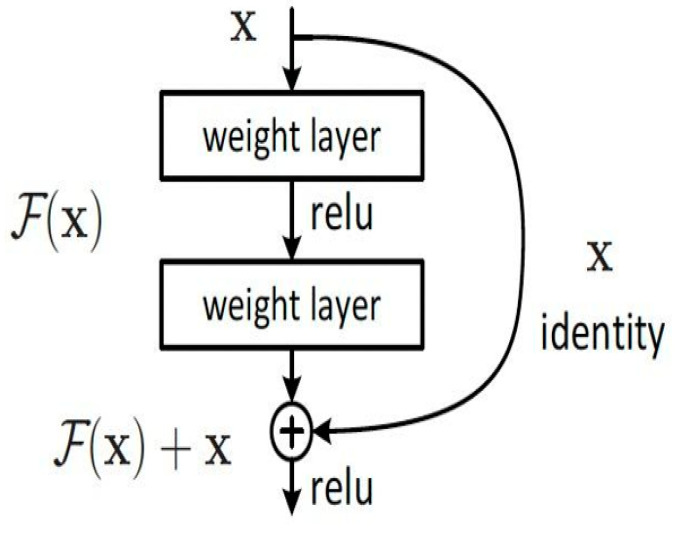
ResNet structure.

The structure of residual learning is shown in Figure 3.

The residual structure proposed in this paper for intrusion detection is described by the following formula. Here, $x_{ij}$ represents the feature vector from the feature matrix of the intrusion detection dataset used for feature extraction by the residual network, and $G_{ij}$ represents the network parameters. As shown in the formula, the next layer of the network can be expressed as the features extracted by the previous layer plus the residual function.

(14) $$x_{i+1j+1}=h\left(x_{ij}\right)+F\left(x_{ij},G_{ij}\right)$$

By analogy, for the features $x_{IJ}$ of a deeper layer in the network, their relationship with the features $x_{ij}$ of a shallow layer can be expressed by the following formula.

(15) $$x_{IJ}=x_{ij}+\sum_{a=1}^{i-1}F\left(x_{a},W_{a}\right)$$

The ResNet module focuses on extracting deep spatial features from the structured network traffic data, effectively learning hierarchical representations of spatial correlations that are indicative of potential intrusions.

### 3.5. AS-TBR Intrusion Detection Model

**Figure 4:**
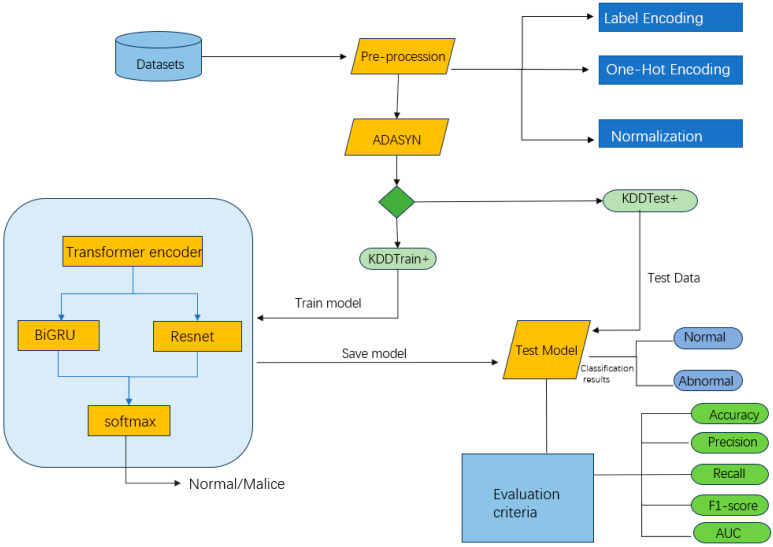
The structure of the network intrusion detection method.

The architecture diagram of the model is shown in Figure 4. The model first preprocesses the input data to ensure consistency in feature representation and improve training effectiveness. The preprocessing steps include one-hot encoding and label encoding for categorical features: one-hot encoding is applied to unordered categories, while label encoding is used for ordered categories to convert them into numerical representations, enhancing the model’s understanding of categorical features.

In addition, numerical features are standardized by scaling them to a standard normal distribution with a mean of 0 and a variance of 1. This eliminates interference caused by differences in scales and accelerates model convergence.

To address the class imbalance issue in the training set (KDDTrain+), the model incorporates Adaptive Synthetic Sampling (ADASYN), which generates minority class samples in sparse regions to dynamically balance the class distribution. The proportion of synthetic samples is set to 40%, increasing the volume of minority class data while avoiding the introduction of excessive redundant samples.

To capture the global temporal dependencies in network traffic data, the model incorporates the encoder part of the Transformer, which has been fine-tuned based on the requirements of the intrusion detection task. Since the goal of intrusion detection is to classify input sequences rather than generate them, the model utilizes only the encoder structure for feature extraction.

The encoder employs a multi-head attention mechanism, which effectively captures long-range dependencies in temporal data and dynamically adjusts the weights of key features. In the feed-forward neural network (FFN) section of the encoder, the model uses a single-hidden-layer perceptron with input and output dimensions being the same. To balance computational complexity and mapping capability, the number of neurons in the hidden layer is set to twice that of the input layer, enhancing the feature mapping ability without significantly increasing computational overhead.

Subsequently, the BiGRU module further models the temporal dependencies in the data, improving the model’s understanding of bidirectional temporal features.

The output features of the BiGRU are fed into a pre-trained ResNet to extract deeper feature representations. By integrating the Transformer encoder, BiGRU, and ResNet modules, the proposed AS-TBR model achieves comprehensive extraction and fusion of spatial and temporal features. This multi-level feature extraction strategy significantly enhances the model’s ability to recognize complex attack patterns by jointly modeling both structural and sequential characteristics of network traffic. Additionally, batch normalization (Batch Normalization) is introduced before the activation layers in ResNet to accelerate model convergence.

## 4. Experimental Setup and Preparation

### 4.1. Dataset

The UNSW-NB15 dataset, released by the University of New South Wales, Australia, is designed for testing and evaluating network intrusion detection systems. This dataset contains approximately 2.5 million records, covering both normal and anomalous (attack) network activities. The records were collected using the IXIA traffic generator (Keysight Technologies, Santa Rosa, CA, USA) on three virtual servers, with two dedicated to normal traffic and the third to anomalous traffic.

The dataset comprises 49 features, which include both packet-based and flow-based characteristics. Packet-based features are extracted from packet headers and payloads, while flow-based features are derived from sequences of packets transmitted between source and destination. The dataset encompasses nine attack types: DoS, Fuzzers, Analysis, Backdoors, Exploits, Generic, Reconnaissance, Shellcode, and Worms.

The NSL-KDD dataset is an improved version of the KDD Cup 1999 network intrusion detection dataset, addressing issues related to redundancy and duplicate records present in the original dataset. By eliminating these redundant data entries, the NSL-KDD dataset enhances representativeness and compactness, making it more suitable for research on network intrusion detection systems. Each data instance consists of 41 features categorized into 4 groups: intrinsic features, content features, time-based features, and host-based features. Data labels include normal traffic (Normal) and various types of attack traffic, which are further classified into four categories: Denial of Service (DoS), Probe, User to Root (U2R), and Remote to Local (R2L) attacks.

### 4.2. Experimental Environment and Parameter Settings

In this experiment, the model was trained and tested in the following hardware and software environments: NVIDIA GeForce RTX 2060 (Nvidia, Santa Clara, CA, USA) was used as the GPU, Intel i7-10087H (Intel, Santa Clara, CA, USA) was used as the CPU, Python 3.8.4 was used as the development language, and PyTorch 2.2.0 was used as the deep learning framework. The dataset was divided into 70% for training, 15% for validation, and 15% for testing. The validation set was used for hyperparameter tuning and model selection, while the testing set was used for the final evaluation of the model. In this experiment, the model was trained and evaluated using the following hyperparameter configurations: a learning rate of 0.001, a batch size of 32, 100 training epochs, and a hidden dimension of 128 for the BiGRU layer. The binary cross-entropy loss function (BCELoss) was used for training, and the Adam optimizer with a learning rate of 0.001 was employed for optimization. These hyperparameter settings were carefully selected to balance the model’s training speed and performance, ensuring its effectiveness when handling imbalanced datasets. The details of the parameter settings are shown in Table 1.

### 4.3. Data Preprocessing

(1) Numericalization

In data preprocessing, numericalization is a necessary step to convert categorical features into numerical formats. This study adopts two methods: one-hot encoding and label encoding. One-hot encoding is applied to unordered categorical features, converting each category into binary features to avoid misinterpretation of ordinal relationships between categories. Label encoding, on the other hand, is used for target labels, mapping categorical labels to integers. In this study, input features are processed using one-hot encoding, while output labels are processed with label encoding to ensure the model can effectively handle both categorical features and labels.

(2) Normalization

In this experiment, standardization was applied to the raw features during data preprocessing to improve the training efficiency and performance of the model. The purpose of data standardization is to scale feature values of different dimensions into the same numerical range, thereby eliminating differences in scales between features. This ensures more balanced weight updates for each feature, accelerates the convergence speed, and avoids problems such as gradient explosion or vanishing caused by large feature value differences.

Specifically, Min-Max normalization was used to scale the value of each feature into the [0, 1] range. The formula is as follows:

(16) $$X^{\prime }=\frac{X-X_{min}}{X_{max}-X_{min}}$$

### 4.4. Data Balancing

To address the class imbalance issue in the dataset, this study implemented the ADASYN scheme, which incorporates an adaptive sampling strategy. Specifically, for the minority class samples that are difficult to classify, the ADASYN method generates synthetic samples to dynamically adjust the distribution of the minority class.

This method generates more synthetic samples in complex classification regions, enhancing the representativeness of these critical samples and aiming to improve the classifier’s ability to recognize the minority class. By focusing on complex regions near the decision boundary, ADASYN optimizes the sample distribution, enabling the model to better learn the boundary between minority and majority classes. Consequently, this approach enhances the classifier’s accuracy and robustness.

### 4.5. Evaluation Metrics

(1) Accuracy

Accuracy refers to the ratio of the number of correctly predicted samples to the total number of samples.

(17) $$Accuracy=\frac{TP+TN}{TP+FP+TN+FN}\times 100\%$$

(2) Precision

Precision refers to the ratio of the number of true positive samples to the total number of samples predicted as positive by the model.

(3) Recall

Recall refers to the ratio of the number of true positive samples successfully predicted as positive by the model to the total number of actual positive samples.

(18) $$Recall=\frac{TP}{TP+FN}\times 100\%$$

(4) F1-Score

The F1-Score is the harmonic mean of precision and recall, comprehensively considering both the precision and recall of the model.

(19) $$F1=2\times \frac{Precision\times Recall}{Precision+Recall}\times 100\%$$

(5) ROC Curve and AUC Value

The ROC (Receiver Operating Characteristic) curve and AUC (Area Under the Curve) are important tools for evaluating the performance of classification models. The ROC curve demonstrates the model’s performance at different thresholds, with the horizontal axis representing the false positive rate (FPR) and the vertical axis representing the true positive rate (TPR). The closer the curve is to the top-left corner, the better the model’s classification performance.

The AUC represents the area under the ROC curve, with values ranging from 0 to 1. The closer the AUC value is to 1, the stronger the model’s ability to distinguish between classes. An AUC of 0.5 indicates that the model performs similarly to random guessing. Generally, an AUC greater than 0.7 is considered to reflect good model performance.

### 4.6. Model Performance

A comprehensive evaluation of the proposed intrusion detection model was conducted on the UNSW-NB15 dataset, covering key metrics such as Precision, Recall, and F1-score. The experimental results (as shown in Table 2) demonstrate that the model exhibits strong performance in identifying both normal and attack traffic. In the “Normal” category, the model achieved a precision of 0.91, a recall of 0.94, and an F1-score of 0.92, indicating high accuracy and strong recall capability in recognizing normal traffic. In the “Attack” category, the model achieved a precision of 0.95, a recall of 0.92, and an F1-score of 0.93, showcasing its robust ability to identify attack traffic. Overall, the model achieved an accuracy of 0.93, with macro-averaged and weighted-averaged precision, recall, and F1-score all reaching 0.93, indicating consistent and efficient performance across different traffic categories. These results further validate that the proposed model effectively balances precision and recall in addressing class imbalance issues, and demonstrates superior performance across multiple evaluation metrics, offering high robustness and practicality.

**Figure 5:**
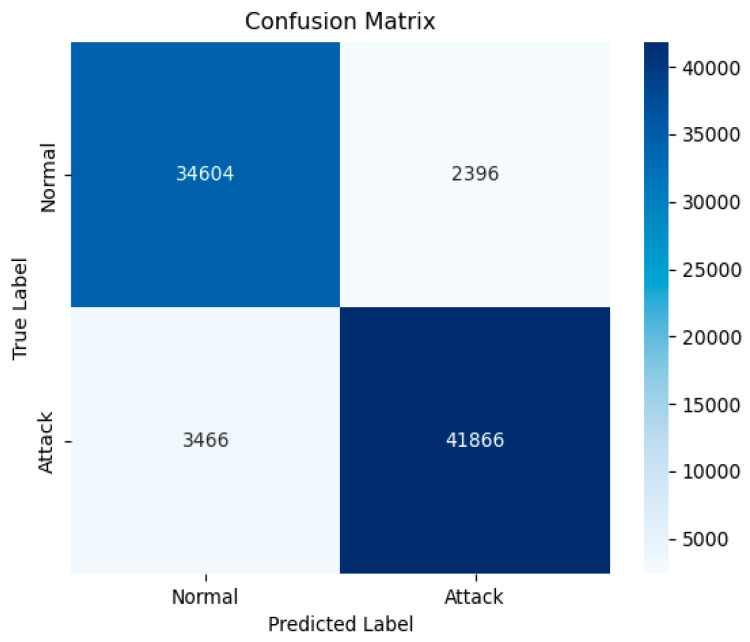
The UNSW-NB15 binary classification confusion matrix.

In the binary classification task on the UNSW-NB15 dataset, the proposed model demonstrated exceptional performance, achieving an AUC value of 0.98, reflecting its outstanding traffic discrimination capability. The confusion matrix (as shown in Figure 5) reveals that the model achieved a precision of 0.93 in the “Normal” category, successfully identifying 34,604 normal traffic samples, with only 2396 misclassified as attack traffic. In the “Attack” category, the recall was 0.92, accurately predicting 41,866 attack traffic samples, while 3466 were incorrectly classified as normal traffic, further validating the model’s effectiveness in distinguishing between the two types of traffic.

**Figure 6:**
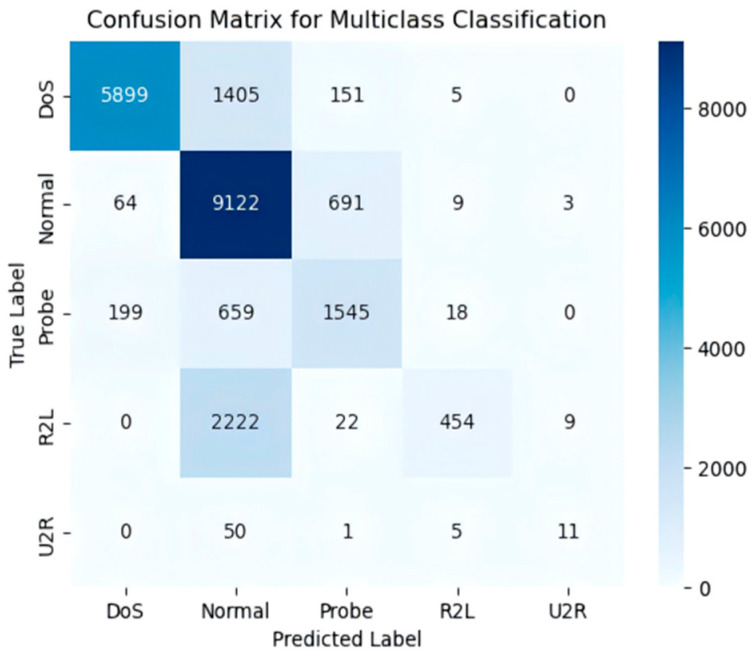
The UNSW-NB15 ten-class classification confusion matrix.

In the multi-classification task, the model also exhibited excellent performance. Data from the confusion matrix (as shown in Figure 6) indicate that the model accurately identified 9122 samples in the “Normal” category, with minimal misclassification. Regarding the AUC values, particularly for the “Generic” category, an impressive AUC of 0.99 was achieved, demonstrating the model’s exceptional ability to recognize this category. Other categories, such as “DoS” and “Reconnaissance,” achieved AUC values of 0.86 and 0.96, respectively, confirming the model’s strong performance in multi-class traffic detection.

**Figure 7:**
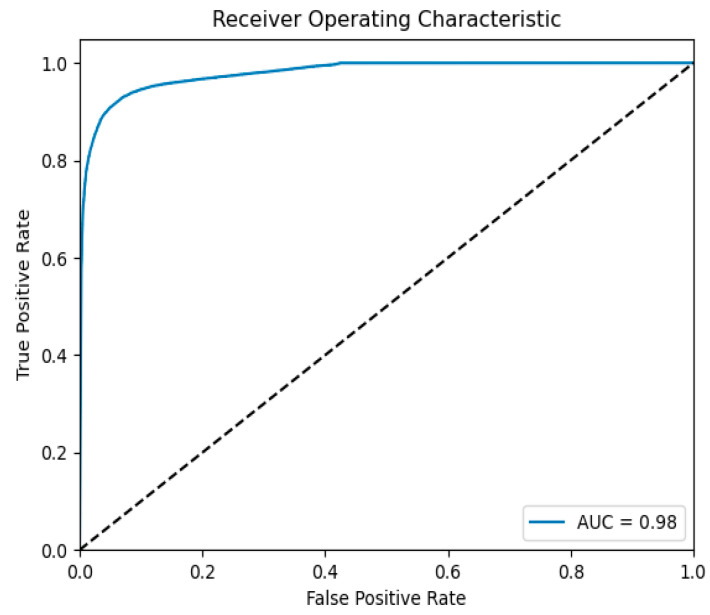
The UNSW-NB15 binary classification ROC curve.

**Figure 8:**
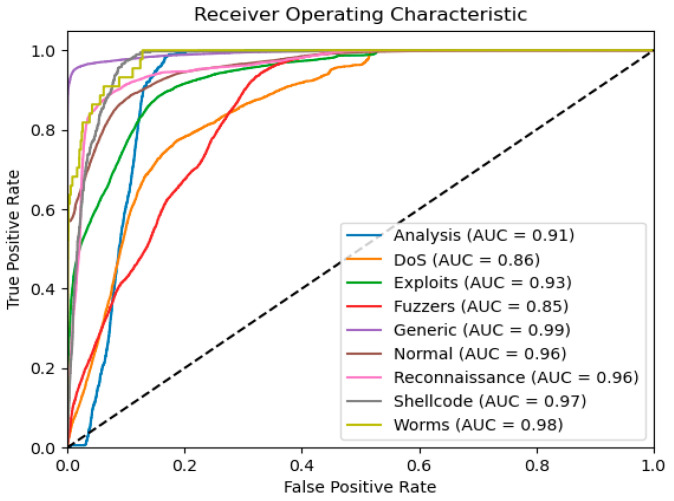
The UNSW-NB15 ten-class classification ROC curve.

The ROC curve further demonstrates the model’s robustness across tasks. In the binary classification task, the AUC value (as shown in Figure 7) was 0.98, fully reflecting the model’s excellent balance between false positive rate and true positive rate. In the multi-classification task, the AUC values (as shown in Figure 8) for all categories exceeded 0.85, with the “Generic” and “Worms” categories achieving 0.99 and 0.98, respectively, indicating that the model maintained strong recognition ability even when handling complex traffic categories.

After evaluating the model on the UNSW-NB15 dataset, we further tested its performance on the NSL-KDD dataset to validate its generalization capability and robustness across different datasets. The performance evaluation results on the NSL-KDD dataset are shown in Table 3. In the “Attack” category, the model achieved a precision of 0.92, a recall of 0.70, and an F1-score of 0.79, indicating high precision and adequate recall ability in detecting attack traffic. In the “Normal” category, the model’s recall was 0.92, precision was 0.70, and the F1-score was 0.79, demonstrating stable performance in recognizing normal traffic. Overall, the model achieved an accuracy of 0.80, with both macro-averaged and weighted-averaged F1-scores of 0.79, reflecting the model’s good balance in handling imbalanced datasets.

**Figure 9:**
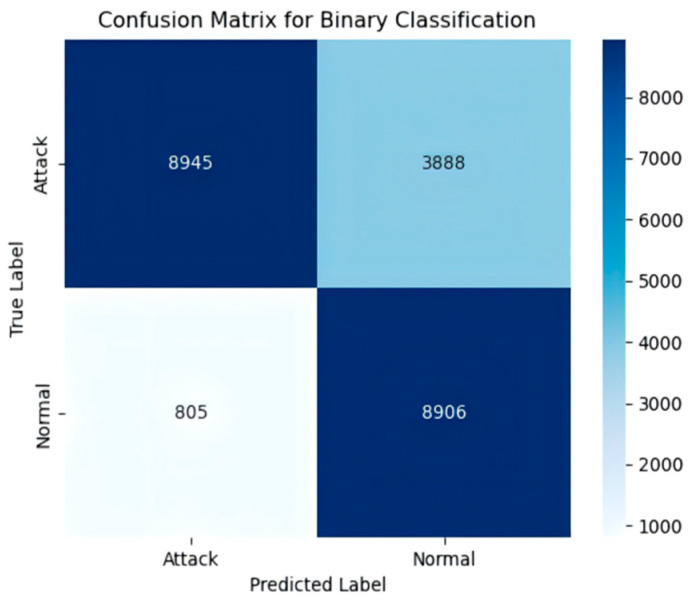
The NSL-KDD binary classification confusion matrix.

In the binary classification task, the confusion matrix (Figure 9) shows that the model correctly classified 8945 “Attack” samples and 8906 “Normal” samples, demonstrating strong recognition capability for both classes.

**Figure 10:**
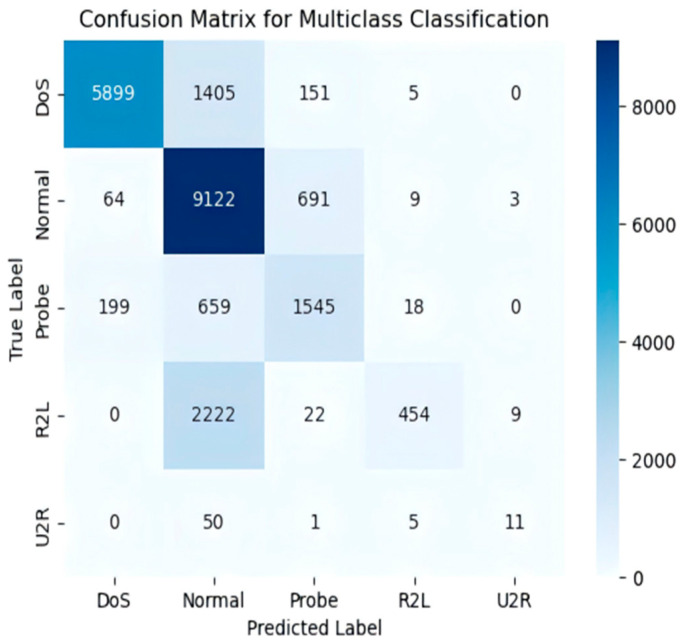
The NSL-KDD five-class classification confusion matrix.

In the multi-class classification task, the confusion matrix (Figure 10) indicates high accuracy for categories such as “DoS” and “Normal.”

**Figure 11:**
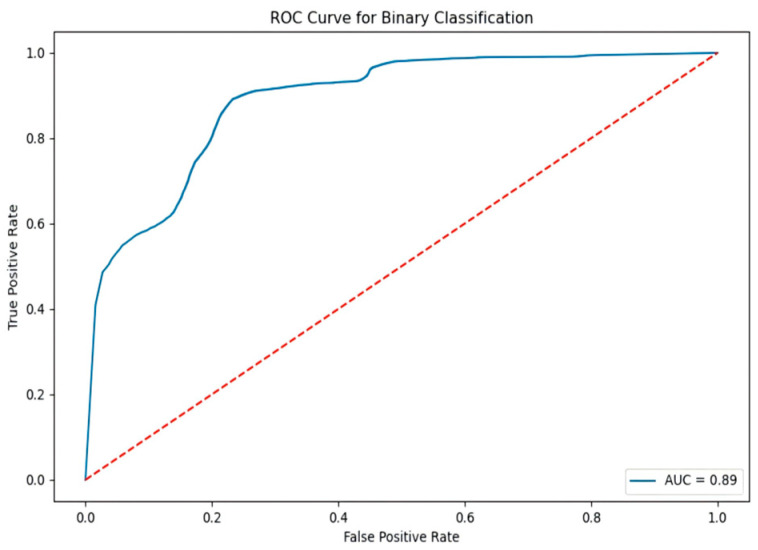
The NSL-KDD binary classification ROC curve.

The ROC curve for the binary task (Figure 11) yields an AUC value of 0.89, indicating the model’s robust classification ability. The curve closely approaches the top-left corner, reflecting low false positive rates and high true positive rates.

**Figure 12:**
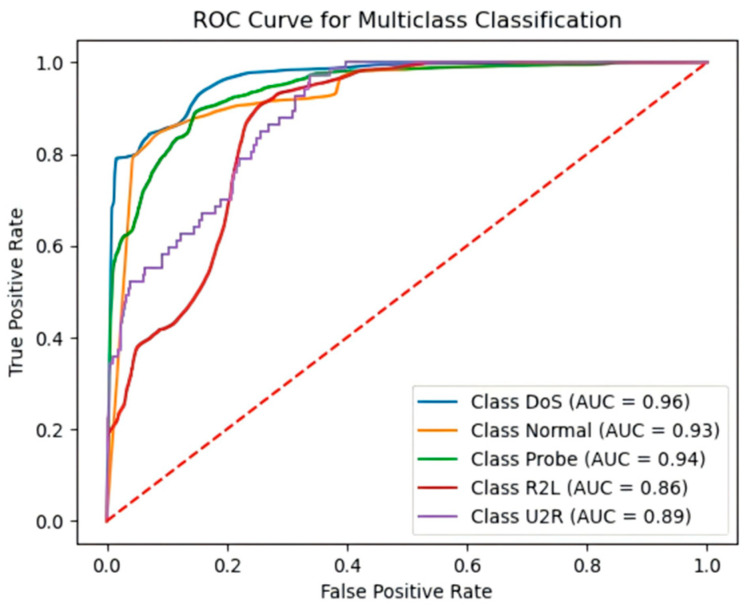
The NSL-KDD five-class classification ROC curve.

For the multi-class task, the ROC curves (Figure 12) show AUC values ranging from 0.86 to 0.96, with the “DoS” category approaching an AUC of 1, further confirming the model’s exceptional performance.

### 4.7. Comparison Between the Proposed Model and Other Models

To evaluate the contribution of each module in the AS-TBR model, we conducted ablation experiments by removing each module and comparing performance changes. The complete model (Transformer + BiGRU + ResNet) achieved the highest accuracy, reaching 93.00%. After removing the Transformer module, the accuracy of the model decreased to 75.55%. After removing the BiGRU module, the accuracy of the model was 84.60%, and after removing the ResNet module, the accuracy of the model was 89.50%. The experimental results show that each module contributes significantly to the performance of the AS-TBR model, and the model that integrates all three modules performs the best, emphasizing the importance of combining the three in achieving optimal performance, as shown in Table 4.

### 4.8. Ablation Experiment

To evaluate the contribution of each module in the AS-TBR model, we conducted ablation experiments by removing each module and comparing performance changes. The complete model (Transformer + BiGRU + ResNet) achieved the highest accuracy, reaching 93.00%. After removing the Transformer module, the accuracy of the model decreased to 75.55%. After removing the BiGRU module, the accuracy of the model was 84.60%, and after removing the ResNet module, the accuracy of the model was 89.50%. The experimental results show that each module contributes significantly to the performance of the AS-TBR model, and the model that integrates all three modules performs the best, emphasizing the importance of combining the three in achieving optimal performance.
ModelAccuracyTransformer + BiGRU + Resnet93.00%BiGRU + Resnet75.55%Transformer + Resnet84.60%Transformer + BiGRU89.50%

## 5. Conclusions

This paper proposes a method based on a hybrid deep learning model (AS-TBR) for addressing the intrusion detection problem in the Advanced Metering Infrastructure (AMI) of smart grids. The model integrates Adaptive Synthetic Sampling (ADASYN) to effectively mitigate the data imbalance issue, thereby significantly enhancing the detection capability for minority class attacks. In terms of feature extraction, this study employs multiple advanced techniques to deeply mine grid data from various dimensions, providing richer information inputs for subsequent intrusion detection. Through comparative experiments, the results show that the AS-TBR model outperforms traditional methods in key metrics such as accuracy and recall rate, particularly demonstrating high robustness in handling minority class attacks. Furthermore, this paper discusses the potential application of the proposed method in real-world smart grid environments, offering both theoretical support and practical implications for the future security of smart grids.

## Figures and Tables

**Table 1:** Parameter settings.

Parameter	Version
Batch Size	32
Epochs	100
Hidden Dim	128
Criterion	BCELoss
Optimizer	Adam
Learning Rate	0.001

**Table 2:** Binary classification performance of the model on the UNSW-NB15 dataset.

Category	Precision	Recall	F1-Score
Attack	0.95	0.92	0.93
Normal	0.91	0.94	0.92
Accuracy			0.93
Macro avg	0.93	0.93	0.93
Weighted avg	0.93	0.93	0.93

**Table 3:** Binary classification performance of the model on the NSL-KDD dataset.

Category	Precision	Recall	F1-Score
Attack	0.92	0.70	0.79
Normal	0.70	0.92	0.79
Accuracy			0.80
Macro avg	0.81	0.81	0.79
Weighted avg	0.81	0.79	0.79

**Table 4:** Comparison of related models.

Reference	Method	Accuracy
[15]	Decision Tree	77.00%
[16]	Random forest	77.00%
[17]	Naive Bayes	76.00%
[18]	CNN-BiLSTM	77.16%
[19]	DNN	91.10%
[20]	LSTM	88.00%
[21]	IGAN	84.45%
[22]	GRU-DAE	91.74%
[23]	CBR-CNN	85.76%
[24]	Tad-GAN	84.26%
AS-TBR	AS-TBR	93.00%

## Data Availability

The datasets generated during this study are not publicly available due to confidentiality agreements with the laboratory. Requests to access the data can be considered on a case-by-case basis by the corresponding author.
